# Substance use and psychotic-like experiences in young people: a systematic review and meta-analysis

**DOI:** 10.1017/S0033291722003440

**Published:** 2023-01

**Authors:** Sandra L. Matheson, Mallory Laurie, Kristin R. Laurens

**Affiliations:** 1 Discipline of Psychiatry and Mental Health, University of New South Wales (UNSW), Sydney, Australia; 2 Neuroscience Research Australia (NeuRA), Sydney, Australia; 3 National Drug and Alcohol Research Centre (NDARC), Sydney, Australia; 4 School of Behavioural and Health Sciences, Australian Catholic University, Brisbane, Australia; 5 School of Psychology and Counselling, Queensland University of Technology (QUT), Brisbane, Australia

**Keywords:** Adolescence, alcohol, cannabis, delusions, hallucinations, illicit drugs, psychosis, psychotic experiences, risk factors, tobacco

## Abstract

This study aimed to systematically review and synthesise the available evidence on the prevalence and associations between psychotic-like experiences (PLEs) and substance use in children and adolescents aged ⩽17 years, prior to the typical age of development of prodromal symptoms of psychosis. As substance use has been associated with earlier age of psychosis onset and more severe illness, identifying risk processes in the premorbid phase of the illness may offer opportunities to prevent the development of prodromal symptoms and psychotic illness. MEDLINE, PsycINFO, and CINAHL databases were searched for chart review, case-control, cohort, twin, and cross-sectional studies. Study reporting was assessed using the Strengthening the Reporting of Observational Studies in Epidemiology (STROBE) checklist, and pooled evidence was evaluated using the Grading of Recommendations Assessment, Development and Evaluation (GRADE) approach. Searches identified 55 studies that met inclusion criteria. Around two-in-five substance users reported PLEs [rate = 0.41, 95% confidence interval (CI) 0.32–0.51; low quality evidence], and one-in-five with PLEs reported using substances (rate = 0.19, 95% CI 0.12–0.28; moderate-to-high quality evidence). Substance users were nearly twice as likely to report PLEs than non-users [odds ratio (OR) 1.77, 95% CI 1.55–2.02; moderate quality evidence], and those with PLEs were twice as likely to use substances than those not reporting PLEs (OR 1.93, 95% CI 1.55–2.41; very low quality evidence). Younger age was associated with greater odds of PLEs in substance users compared to non-users. Young substance users may represent a subclinical at-risk group for psychosis. Developing early detection and intervention for both substance use and PLEs may reduce long-term adverse outcomes.

## Introduction

Substance use is an established risk factor for earlier and more severe psychotic outcomes (Andrade, 2016; Helle et al., 2016). Initiation of use typically occurs during adolescence, when the developing brain is especially vulnerable to the deleterious effects of substances (Degenhardt, Stockings, Patton, Hall, & Lynskey, 2016; Gururajan, Manning, Klug, & van den Buuse, 2012). Exposure to substances may be particularly detrimental for young people who present risk factors for psychosis (Kelleher et al., 2012), with substance use interventions delivered to youth at high-risk of psychosis highlighted as a potential avenue for the prevention of psychotic disorders (Carney, Cotter, Firth, Bradshaw, & Yung, 2017).

Previous reviews examining the relationship between substance use and psychosis have focused predominantly on cannabis and clinical psychosis outcomes. Among these, a systematic review indicated that cannabis use prior to the age of 18 years increased risk of an earlier onset of psychosis only among cases with more severe use and pre-existing vulnerability – that is, a family history of psychosis (Bagot, Milin, & Kaminer, 2015). Subsequent meta-analyses have described a dose–response relationship of increasing likelihood of psychosis with increasing use of cannabis (Marconi, Di Forti, Lewis, Murray, & Vassos, 2016), and reported that adolescent cannabis use increased the risk for psychosis and predicted an earlier onset of the disorder, with family history of psychosis, earlier age of onset and frequency of cannabis use, and concurrent use of other substances all strengthening the association (Kiburi, Molebatsi, Ntlantsana, & Lynskey, 2021). Another meta-analysis identified the age at onset of psychosis for cannabis users as 2.7 years younger than for non-users, and for those with broadly defined substance use, the age at onset of psychosis was 2.0 years younger (Large, Sharma, Compton, Slade, & Nielssen, 2011).

Concurring effects have been described among young people at clinical high-risk (CHR) of psychosis who are putatively in the prodromal phase of illness that immediately precedes the onset of frank psychosis. In a meta-analysis in which the majority of CHR individuals experienced attenuated or brief intermittent psychotic symptoms (Carney et al., 2017), compared to non-CHR controls, CHR individuals had higher rates of cannabis use (27% *v.* 17%) and cannabis use disorders, and CHR cannabis users experienced more severe psychotic symptoms than CHR non-users. In another meta-analysis, current (but not lifetime) cannabis use disorder increased risk of psychosis among CHR youth (Kraan et al., 2016).

In terms of substance use more broadly, a meta-analysis examining relationships between a range of environmental risk factors and subclinical psychotic experiences in child and adult samples identified the use of cannabis, alcohol, as well as other substances, as risk factors for later psychotic experiences (Linscott & van Os, 2013). Meta-analyses have also described a significant association between CHR state and tobacco use (Fusar-Poli et al., 2017), with 33% of CHR individuals smoking tobacco relative to 14% of non-CHR controls (Carney, Cotter, Bradshaw, Firth, & Yung, 2016).

It remains unclear whether substance use and psychotic experiences relate to each other as causal, triggering, or maintaining factors. However, robust data from randomised and controlled laboratory studies suggest that exposure to substances such as cannabis causes disruptions to brain development that elicit negative psychiatric outcomes (Sherif, Radhakrishnan, D'Souza, & Ranganathan, 2016). Intravenous delta-9-tetrahydrocannabinol administration (Δ-^9^-THC; the active ingredient of cannabis that causes the psychoactive effects) has been found to have a dose-dependent effect on psychotic-like symptoms in healthy volunteers (D'Souza et al., 2004). Studies of rodents have found psychotic-like signs in adult rodents after adolescent cannabinoid exposure, but not after adult cannabinoid exposure (Rubino & Parolaro, 2014), suggesting adolescence constitutes a more vulnerable exposure window.

The aim of this systematic review was to assess the current evidence relating to both the prevalence of any substance use (including tobacco, alcohol, cannabis, and other substances) in children and adolescents who report experiencing subclinical psychotic symptoms (or psychotic-like experiences; PLEs), and the prevalence of PLEs in those who report using substances. We further sought to compare these prevalence rates to those in comparison (control) groups. An upper age limit of 17 years was chosen in order to restrict the analyses to studies focused on the period prior to the typical age of onset of the psychosis prodrome during later adolescence or young adulthood (Ruhrmann, Schultze-Lutter, & Klosterkötter, 2010; Tandon, Nasrallah, & Keshavan, 2009; Yung et al., 2009). We restricted our analyses to this pre-prodrome period to identify prospects for earlier intervention. Among CHR individuals, more than one-in-five (22%) transition to psychotic illness within 3 years (Fusar-Poli et al., 2020), and many experience persistent psychopathology, psychosocial impairment, and poor quality of life (Simon et al., 2013). These outcomes highlight the need for earlier detection and intervention to prevent prodromal symptoms and their associated adverse outcomes (Laurens & Cullen, 2016).

## Method

The review was registered with PROSPERO (CRD42018106597) and conducted according to PRISMA (Preferred Reporting Items for Systematic Reviews and Meta-Analyses) guidelines (Moher, Liberati, Tetzlaff, & Altman, 2009). All exclusion/inclusion decision-making, data extraction, and data analyses were performed in duplicate by two authors (SLM, and ML or KRL), with any disagreements resolved by discussion between authors.

### Study eligibility and search strategy

The review incorporated cross-sectional, cohort, twin, and case-control studies. Inclusion criteria were: (1) studies of participants aged ⩽17 years; and (2) studies that reported assessment of both PLEs and substance use in at least 75% of participants, as measured by self-/informant-report questionnaires, interviews, or case notes. Exclusion criteria were: (1) studies of participants with a diagnosis of psychotic illness and (2) a lack of primary data (e.g. reviews). Searches were conducted in MEDLINE, PsycINFO, and CINAHL to identify articles published in English until July 2022 (updating the initial scoping search conducted in April 2018). Search terms are detailed in online Supplementary materials (S1). Articles were screened for eligibility in three stages: (1) by title and abstract; (2) by full-text review; and (3) by manual search of the reference lists of the eligible articles to locate studies not identified by database search.

### Data extraction

The following information was extracted from the included studies: (1) study characteristics, including study design and setting; (2) sample characteristics, including sample size, mean age, and gender (% male); (3) substance use characteristics, including, where available, the type of substance used, if assessed when intoxicated, and the assessment method and tool used; (4) PLE characteristics, including type of PLE, the assessment method, and tool used; and (5) counts of adolescents with and without PLEs/substance use or, if no counts were reported, measures of association were extracted.

### Summary measures and synthesis of results

Studies were categorised into four meta-analyses: prevalence of PLEs in youth with substance use (meta-analysis 1a); comparison of the prevalence of PLEs in youth with *v.* without substance use (meta-analysis 1b); prevalence of substance use in youth with PLEs (meta-analysis 2a); and comparison of the prevalence of substance use in youth with *v.* without PLEs (meta-analysis 2b). Studies were allocated to each meta-analysis according to the data available; studies reporting raw data for cases only were allocated to meta-analyses 1a and/or 2a, studies reporting raw data for both cases and controls were allocated to meta-analyses 1a, 1b, 2a, and/or 2b, while studies reporting only effect sizes were allocated to meta-analyses 1b and/or 2b. Given sufficient data, additional meta-analyses were conducted on correlations to assess dose-dependence between substance use and PLEs. To ensure independence of the main analyses, if studies reported data on multiple time frames, PLEs, or substance use types in the same participants, data from the most commonly reported PLE or substance across studies only was used.

Meta-analyses were completed in Comprehensive Meta-Analysis Version 3 [CMA-3; Borenstein, Hedge, Higgins, & Rothstein, 2009] using random effects models. For meta-analyses 1a and 2a (prevalence studies), pooled data were compiled as event rates, and for meta-analyses 1b and 2b (association studies), odds ratios (ORs) and their 95% confidence intervals (CIs) were calculated. We pooled count data, ORs and their CIs, with means and their standard deviations (s.d.s) for meta-analyses 1b and 2b. Where studies reported only risk ratios for a PLE outcome, these were treated as ORs (Zhang & Yu, 1998) in studies of adolescents, as the prevalence of PLEs is <10% in this population (13–18 years; 7.5%; Kelleher et al., 2012). This was not done in studies of children, as PLEs are more common in that age group (9–12 years; 17%; Kelleher et al., 2012). The reverse was true for substance use outcome, as adolescence is the peak period during which substance use occurs (Degenhardt et al., 2016).

Effect sizes for ORs were defined as small if OR < 2.0, medium if OR between 2.0 and 5.0, large if OR > 5.0, and very large if OR > 10.0. Differences in percentages were defined as small if ~7, medium if ~18, large if ~30, and very large if ⩾45, and effect sizes for correlations were weak if *r* ~ 0.10, medium if *r* ~ 0.30, and strong if *r* ⩾ 0.50 (Rosenthal, 1996). Heterogeneity was measured with the *Q* test and *I*^2^, where the *I*^2^ statistic indexes the percentage of the variability in effect estimates that is due to heterogeneity rather than to sampling error. Outliers and influencers were assessed using the one-study removed analysis (Viechtbauer & Cheung, 2010). Funnel-plot analyses assessed risk of publication bias. Where Egger's test indicated possible publication bias, Duval and Tweedie's trim and fill test was reported, which provides an adjusted effect size for a symmetric funnel plot (Borenstein et al., 2009).

Given sufficient studies, planned subgroup and meta-regression analyses were conducted to assess causes of heterogeneity. Potential moderators included: study quality, gender distribution, age at assessment, method of assessment, frequency of substance use, type of substance use, type of PLE, whether the PLE occurred while intoxicated, and whether the ORs were adjusted. For subgroup analyses, we did not assume a common variance within subgroups, so results may be imprecise in analyses that included fewer than five studies in each subgroup (Borenstein et al., 2009). Meta-regressions were conducted with a restricted maximum likelihood model, as recommended for small-to-medium-sized meta-analyses, and the Knapp Hartung distribution, recommended for random effects models (Borenstein et al., 2009). When statistical pooling was not possible, relevant studies were retained for narrative reporting.

### Quality assessments

A standardised critical appraisal instrument [the Strengthening the Reporting of Observational Studies in Epidemiology (STROBE) checklist; www.strobe-statement.org] was used to assess included study reporting quality. A percentage score was calculated for each study to represent the total number of STROBE items reported. Percentage scores were then averaged across studies in each meta-analysis to give an indication of overall risk of study bias, with averaged scores ⩽25% rated as high risk of study bias, and ⩾75% rated as low risk of study bias.

The quality of the pooled evidence was evaluated using the Grading of Recommendations, Assessment, Development and Evaluation (GRADE) approach. According to GRADE, pooled results from observational studies are considered inherently of low quality due to possible confounding factors which should be evenly distributed across groups in randomised studies. Quality of results can be upgraded if overall risk of study bias is low, if samples are large, if pooled effect sizes are large or dose dependent, if there is no residual confounding, or if the evidence is direct, consistent, or precise (Guyatt et al., 2011). Indirectness refers to approximated measures, comparisons, or samples; inconsistency to significant heterogeneity among studies results; and imprecision to large CIs across the pooled effect size (CIs > 0.25 in either direction; Schunemann, 2008). As GRADE guidelines for measuring precision do not apply to prevalence data, we considered pooled event rates as imprecise if CIs were larger than 10% in either direction.

## Results

### Study selection

**Fig. 1.**
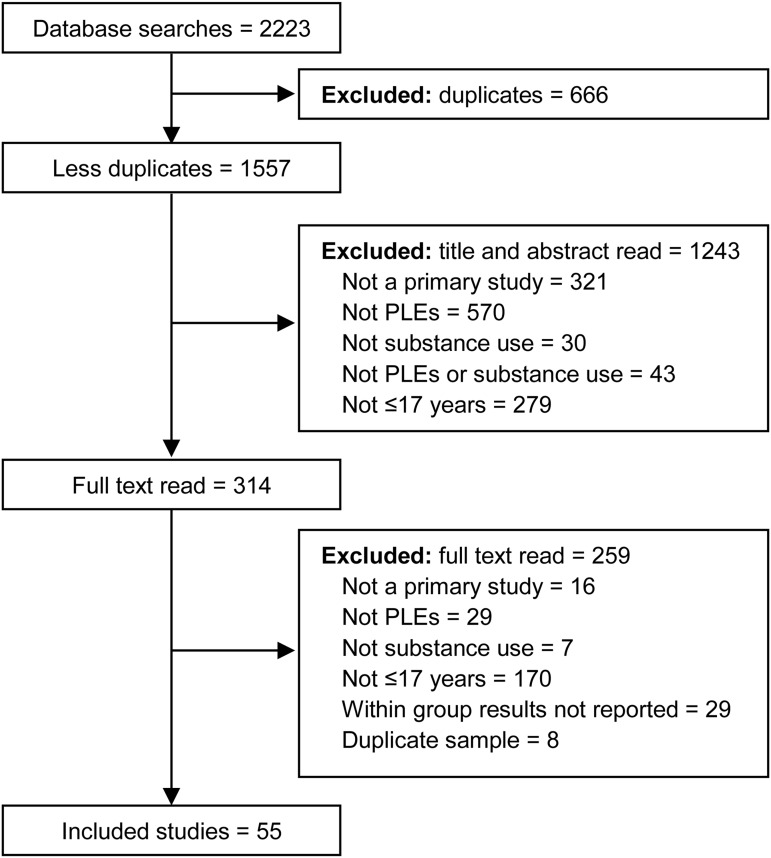
Flow diagram showing the process of inclusion/exclusion through the different phases of the meta-analysis.

As detailed in the flow diagram illustrated in Fig. 1, the searches yielded 2223 references, of which 666 were excluded as duplicates, 1243 were excluded following review of title and abstract, and a further 259 were excluded following full text review. The remaining 55 studies met inclusion criteria (Addington et al., 2019; Albertella & Norberg, 2012; Auther et al., 2012; Barkhuizen, Taylor, Freeman, & Ronald, 2019; Bassett, Schunk, & Crouch, 1996; Bechtold, Hipwell, Lewis, Loeber, & Pardini, 2016; Besli, Ikiz, Yildirim, & Saltik, 2015; Bourque, Afzali, O'Leary-Barrett, & Conrod, 2017a; Bourque et al., 2017b; Brink et al., 2020; Colins, Vermeiren, Noom, & Broekaert, 2013; Colins et al., 2009; Cruz & Domínguez, 2011; DaBreo-Otero, 2021; Dolphin, Dooley, & Fitzgerald, 2015; Drobinin et al., 2020; Evans & Raistrick, 1987; Fonseca-Pedrero, Lucas-Molina, Pérez-Albéniz, Inchausti, & Ortuño-Sierra, 2020; Forrester, 2012; Friedman, Utada, Glickman, & Morrissey, 1987; Garland & Howard, 2010; Goulter, McMahon, & Dodge, 2019; Harley et al., 2010; Hartsell, 2021; Hides et al., 2009; Jones, Calkins, Scott, Bach, & Gur, 2017; Jones, Gage, & Heron, 2018; Konings, Henquet, Maharajh, Hutchinson, & Van Os, 2008; Lansing, Plante, Fennema-Notestine, Golshan, & Beck, 2018; Levy & Weitzman, 2019; Lindgren et al., 2010; Mackie, Castellanos-Ryan, & Conrod, 2011; Mackie et al., 2021; McGorry et al., 1995; McMahon et al., 2021; Miettunen et al., 2008; Mundy, Robertson, Robertson, & Greenblatt, 1990; Opaleye et al., 2009; Rimvall et al., 2020; Schifano, Forza, & Gallimberti, 1994; Scott et al., 2009; Shakoor et al., 2015; Shervette, Schydlower, Lampe, & Fearnow, 1979; Shrier, Harris, Kurland, & Knight, 2003; Stain et al., 2016; Stainton et al., 2021; Sunderland et al., 2021; Tekulve, Alexander, & Tormoehlen, 2014; van Gastel et al., 2012; Vaughn, 2006; Wang et al., 2022; Watts et al., 2021; Whitt, Garland, & Howard, 2012; Yilmaz Kafali et al., 2022; Zammit, Owen, Evans, Heron, & Lewis, 2011). Table 1 summarises the characteristics of the included studies, and online Supplementary Table S2 details each STROBE item rating.

**Table 1.** Descriptive summary of the included studies

Study ID	Study design	Setting	Sample characteristics	Substance use assessment	PLE assessment	Meta-analyses	STROBE (%)
Addington 2019	Cohort	Mental health facilities, Canada	*N* = 108, 43.5% male, age = 16.8	Interview: AUS, DUS, alcohol and cannabis	Interview: SIPS, any PLE	Yes, 2a	96
Albertella 2012	Pre-post treatment	Residential substance treatment facility, Australia	*N* = 132, 76.5% male, age = 16.7	Interview: The Severity of Dependence Scale modified for cannabis use	Interview: BSI, paranoid ideation and psychoticism	No, beta coefficients	76
Auther 2012	Cohort	Health care facilities and community, USA	*N* = 160, 60.0% male, age = 16.1	Interview: K-SADS-E, any substance	Interview: SOPS, any PLE	Yes, 2a, 2b	84
Barkhuizen 2019	Twin study	Community, UK	*N* = 3787, 46% male, age = 16.2	Self-report: tobacco	Self-report: Specific Psychotic Experiences Questionnaire, paranoia and hallucinations	Yes, 1b	91
Bassett 1996	Chart review	Health care facilities, USA	*N* = 80, 59% male, age = 15.0	Chart review: cyclizine hydrochloride	Chart review: hallucinations while intoxicated	Yes, 1a	68
Bechtold 2016	Cross-sectional	Secondary schools, USA	*N* = 888, 100% male, age = 13–18	Self-report: SUQ, cannabis	Self-report: YSR, paranoia, hallucinations, and bizarre thinking	Yes, 1b, 2b	94
Besli 2015	Chart review	Emergency departments, Turkey	*N* = 16, 94% male, age = 15.4	Chart review: synthetic cannabis	Interview: hallucinations and perceptual changes while intoxicated	Yes, 1a	67
Bourque 2017a	Cohort	Secondary schools, Canada	*N* = 2566, 50.4% male, age = 15.8	Self-report: alcohol, cannabis, and tobacco	Self-report: PLEQ-C, hallucinations, and delusions	Yes, 2a, 2b	90
Bourque 2017b	Cohort	Secondary schools, UK	*N* = 162, 35% male, age = 14.3	Self-report: alcohol, cannabis, and tobacco	Interview: APSS, perceptual abnormalities and delusional thoughts	Yes, 2b	87
Brink 2020	Cohort	Secondary schools, The Netherlands	*N* = 123, 37.4% male, age = 13.6	Self-report: Social and Health Assessment Scale, alcohol and cannabis	Self-report: PQ, hallucinations	Yes, 2a	97
Colins 2009	Cross-sectional	Youth detention centres, Belgium	*N* = 231, 100% male, age = 16.0	Interview: DISC-IV, cannabis, amphetamines, and cocaine	Interview: DISC-IV, delusions, hallucinations, and catatonia	Yes, 2a, 2b	87
Colins 2013	Cohort	Detention centres, Belgium	*N* = 224, 100% male, age = 16.5	Interview: DISC-IV, any substance	Interview: DISC-IV, hallucinations and delusions	Yes, 2a	85
Cruz 2011	Cross-sectional	Secondary schools, Mexico	*N* = 17, 59% male, age = 15.3	Interview: toluene-based solvent	Interview: hallucinations while intoxicated	Yes, 1a	81
DaBreo Otero 2021	Cohort	Prevention programme, USA	*N* = 156, 70.5% male, age = 15.9	Interview: K-SADS-E, any substance	Interview: SOPS, any PLE	Yes, 2a, correlation with substance use outcome	81
Dolphin 2015	Cross-sectional	Secondary schools, Ireland	*N* = 5910, 49% male, age = 14.9	Interview: AUDIT, cannabis, alcohol, and other substances	Interview: APSS, auditory, visual hallucinations, and paranoia	Yes, 2b	90
Drobinin 2020	Cross-sectional	Nova Scotia health settings, Canada	*N* = 110, 42% male, age = 14.0	Interview: cannabis, stimulants	Interview: K-SADS-PL, SIDS, and SPI-CY, any PLE	Yes, 2a, 2b	92
Evans 1987	Cross-sectional	Substance use treatment facility, UK	*N* = 43, % male not reported, age = 15.7	Interview: inhalants	Interview: hallucinations and delusions while intoxicated	Yes, 1a	52
Fonseca-Pedrero 2020	Cross-sectional	Secondary schools, Spain	*N* = 1588, 46.5% male, age = 16.1	Interview: Modified Substance Use Questionnaire, cannabis	Self-report: PQ-B, any PLE	Yes, 1a, 1b, 2a; 2b	86
Forrester 2012	Chart review	Texas Poison Centre Network, USA	*N* = 305, 72% male, age = 16.7	Chart review: synthetic cannabis	Chart review: any PLE	Yes, 2a	87
Friedman 1987	Cohort	Secondary schools, USA	*N* = 232, 55% male, age = 15.1	Self-report: alcohol, cannabis, inhalants, amphetamines, and other substances at follow-up	Self-report: BSI, paranoid ideation, and psychoticism at baseline	No, PLE means *v.* BSI norms	70
Garland 2010	Cross-sectional	Residential forensic custody, USA	*N* = 267, 84.6% male, age = 15.5	Interview: inhalants	Interview: visual and auditory hallucinations while intoxicated	Yes, 1a	80
Goulter 2019	Cohort	Elementary schools, USA	*N* = 891, 69% male, age = 6–16	Self-report: Things That You Have Done and the Tobacco, Alcohol and Drugs survey, cannabis	Parent-report: CBCL, thought problems	Yes, correlation with substance use outcome	75
Harley 2010	Case-control	Secondary schools, Ireland	*N* = 195, % male not reported, age = 12–15	Interview: K-SADS-PL, cannabis	Interview: K-SADS-PL, hallucinations	Yes, 1a; 1b	84
Hartsell 2021	Cohort	Juvenile offenders, USA	*N* = 1354, 82% male, age = 16–16.5	Self-report: cannabis	Self-report: BSI, any PLE	Yes, 2b	83
Hides 2009	Cross-sectional	Secondary schools, Australia	*N* = 880, 46.9% male, age = 15.0	Self-report: YRBS, cannabis	Self-report: any PLE	Yes, 1b	83
Jones 2017	Cohort	Community, USA	*N* = 4208, 45% male, age = 16.9	Self-report: cannabis, alcohol, and tobacco	Interview: K-SADS-PL, SOPS, any PLE	Yes, 1b, 2a, 2b	90
Jones 2018	Cohort	Community birth cohort, UK	*N* = 5300, 43.9% male, age = 16.0	Interview: tobacco and cannabis	Interview: PLIKSi, any PLE	Yes, 2b	91
Konings 2008	Cross-sectional	Secondary schools, Trinidad	*N* = 431, 45% male, age = 16.0	Self-report: cannabis	Self-report: CAPE, any PLE	No, beta coefficients	85
Lansing 2018	Cross-sectional	Rehabilitation facility, USA	*N* = 158, 0% male, age = 16.0	Interview: Customary Drug Use Record Form, cannabis, opioids, and prescription medications	Interview: SCI-PSY, paranoia, delusions, and hallucinations	Yes, 1a, correlation with PLE outcome	93
Levy 2019	Cross-sectional	Routine care setting, USA	*N* = 146, 22.8% male, age = 16.6	Interview: modified CIDI, cannabis	Self-report: hallucinations and paranoia during and after intoxication	Yes, 1a	81
Lindgren 2010	Case-control	Adolescent psychiatric facilities, Finland	*N* = 174, 28.8% male, age = 16.6	Interview: Structured Clinical Interview for the DSM-IV, any substance	Interview: SIPS, any PLE	Yes, 2a, 2b	81
Mackie 2011	Cohort	Secondary schools, UK	*N* = 409, 34% male, age = 14.7	Self-report: Reckless Behaviour Questionnaire, any substance	Self-report: PLEQ-C, any PLE	Yes, 2a, 2b	93
Mackie 2021	Cross-sectional	Secondary schools, UK	*N* = 567, 56.1% male, age = 16.8	Self-report: Smoking Drinking and Drug use Questionnaire, MMM, cannabis	Self-report: SPEQ, hallucinations and paranoia	Yes, 1a, 1b	93
McGorry 1995	Cross-sectional	Secondary schools, Australia	*N* = 657, 43.8% male, age = 16.5	Self-report: Adolescent Health Survey, cannabis, amphetamine, and other substances	Self-report: Adolescent Health Survey, magical ideation, and perceptual disturbances	Yes, correlation with PLE outcome	86
McMahon 2021	Cross-sectional	Secondary schools, Ireland	*N* = 973, 53.6% male, age = 14.7	Self-report: alcohol, tobacco, cannabis	Self-report: Adolescent Psychotic Symptom Screener, any PLE	Yes, 2a, 2b	93
Miettunen 2008	Cohort	Community setting, Finland	*N* = 6298, 48.3% male, age = 15–16	Self-report: cannabis	Self-report: PROD-screen, any PLE	Yes, 1a, 1b	80
Mundy 1990	Cross-sectional	Homeless adolescents, USA	*N* = 96, 61% male, age = 16.1	Interview: HAIS, drugs and alcohol	Interview: HAIS, hallucinations, paranoia, ideas of reference	Yes, correlation with PLE outcome	88
Opaleye 2009	Cross-sectional	Homeless adolescents, Brazil	*N* = 78, 79% male, age = 9–18	Interview: benzydamine	Interview: hallucinations	Yes, 1a	92
Rimval 2020	Cohort	Birth cohort, Denmark	*N* = 1138, 45.2% male, age = 16.0	Interview: ASSIST, any substance	Interview: PLIKS-Q, any PLE	Yes, 2b	83
Schifano 1994	Cross-sectional	Secondary schools, Italy	*N* = 564, 0% male, age = 14–17	Self-report: tobacco	Self-report: SCL-90, paranoid ideation and psychoticism	Yes, 1b	83
Scott 2009	Cross-sectional	Community, Australia	*N* = 1261, 47.8% male, age = 14.8	Self-report: YRBS, cannabis and alcohol	Self-report: YSR, auditory and visual hallucinations	Yes, 2b	80
Shakoor 2015	Twin study	Community setting, UK	*N* = 9660, 45% male, age = 16.3	Self-report: cannabis	Self-report: SPEQ, paranoia and hallucinations	Yes, 1b	90
Shervette 1979	Chart review	Medical centre, USA	*N* = 29, 76% male, age = 16.1	Chart review: Jimson weed	Chart review: hallucinations while intoxicated	Yes, 1a	46
Shrier 2003	Cross-sectional	Primary care facility, USA	*N* = 538, 32% male, age = 16.6	Self-report: POSIT, any substance	Interview: ADI, hallucinations and delusions	Yes, 1a, 1b	70
Stain 2016	RCT	Community mental health facilities, Australia	*N* = 57, 42% male, age = 15.6	Interview: OTI, Alcohol and Cannabis Use Disorders Identification Tests, Severity of Dependence Scale	Interview: CAARMS, any PLE	Yes, 2a	100
Stainton 2021	Cross-sectional	Secondary schools, UK	*N* = 302, 40% male, age = 15.6	Self-report: cannabis	Self-report: CAPE, any PLE	Yes, 1b	79
Sunderland 2021	Cross-sectional	Household survey, Australia	*N* = 2003, 51.4% male, age = 15.5	Self-report: cannabis	Self-report: any PLE	Yes, correlation with substance use outcome	85
Tekulve 2014	Chart review	Health care facilities, USA	*N* = 1328, 70.5% male, age = 17.0	Chart review: synthetic cathinone	Chart review: hallucinations and delusions during intoxication	Yes, 1a	53
van Gastel 2012	Cross-sectional	Secondary schools, The Netherlands	*N* = 4552, 50% male, age = 12–16	Self-report: cannabis	Self-report: CAPE, all PLEs	Yes, 1b	77
Vaughn 2006	Cross-sectional	Detention centre, USA	*N* = 728, 87% male, age = 15.5	Interview: MAYSI-II, any illicit substance	Interview: BSI, paranoid ideation	No, beta coefficients	86
Wang 2022	Cross-sectional	Junior and senior schools, China	*N* = 67 532, 53.7% male, age = 14.6	Self-report: survey, alcohol and tobacco	Self-report: CAPE, any PLE	Yes, 1b	96
Watts 2021	Cross-sectional	Elementary schools, USA	*N* = 11 872, 47% male, age = 10.0	Self-report: iSay Sip Inventory, alcohol	Self-report: PQ-B, any PLE	Yes, 2b	73
Whitt 2012	Cross-sectional	Residential rehabilitation facility, USA	*N* = 723, 87% male, age = 15.5	Interview: VSSI, inhalant	Interview: BSI, psychoticism, paranoid ideation, and hallucinations	Yes, 1b	76
Yilmaz 2022	Case-control	Children's hospital, Turkey	*N* = 684, 48% male, age = 15.9	Self-report: any substance	Self-report: CAPE	Yes, 1b	96
Zammit 2011	Cohort	Community, UK	*N* = 2630, % male not reported, age = 14–16	Self-report: cannabis	Interview: PLIKS-Q, hallucinations and delusions	Yes, 1a, 1b, 2a, 2b	94

ADI, Adolescent Diagnostic Interview; APSS, Adolescent Psychotic Like Symptoms Screener; ASSIST, Alcohol, Smoking and Substance Involvement Screening Test; AUDIT, Alcohol Use Disorders Identification Test; BSI, Brief Symptom Inventory; CAPE, Community Assessment of Psychotic Experiences; CAARMS, Comprehensive Assessment of At Risk Mental States; CBCL, Child Behaviour Checklist; CIDI, Composite International Diagnostic Interview; DISC-IV, Diagnostic Interview Schedule for Children, version 5; HAIS, Homeless Adolescent Interview Schedule; K DISC-IV, Diagnostic Interview Schedule for Children, Fourth Edition; K-SADS-PL, The Kiddie Schedule for Affective Disorders and Schizophrenia Present and Lifetime; MAYSI-II, Massachusetts Youth Screening Instrument Second Version, Inventory; MMM, Marijuana Motives Measure; OTI, Opiate Treatment Index; PLE, psychotic-like experience; PLEQ-C = Psychotic-Like Experiences Questionnaire for Children; PLIKSi, Psychosis-like Symptom Interview; PLIKS-Q = Psychotic-Like Symptoms Questionnaire; POSIT, Problem Oriented Screening Instrument for Teenagers; PQ, Prodromal Questionnaire; PQ-B, Prodromal Questionnaire – Brief Version; PROD-screen, screen for prodromal symptoms of psychosis; RCT, randomised controlled trial; SADS-E, The Kiddie Schedule for Affective Disorders and Schizophrenia Epidemiological; SCI-PSY, Clinical Interview for Psychotic Spectrum; SCL-90, Symptom Checklist-90-Revised; SIDS, Structured Interview for Prodromal Syndromes; SOPS, Scale of Prodromal Symptoms; SPEQ, Specific Psychotic Experiences Questionnaire; SPI-CY, Schizophrenia Proneness Instrument – Child and Youth version; SUQ, Substance Use Questionnaire; VSSI, Volatile Solvent Screening, YSR, Youth Self Report; YRBQ, Youth Risk Behaviour Survey.

An overall summary of the results and GRADE quality assessments associated with each primary meta-analysis is provided in Table 2.

**Table 2.** Primary meta-analyses results and GRADE quality assessments

No. of studies, mean STROBE (%)^a^	Total sample^b^	Effect size^c^, *p* value	Precision^d^ 95% CI	Consistency^e^ *I*^2^, *Qp* value	Directness^f^	Residual confounding^g^	Publication bias^h^	Quality rating
Meta-analysis 1a: event rates of PLEs in adolescents with substance use								
16, 76%^↑^	3050↑	ER = 0.41^↑^, *p* = NA	0.32–0.51^↓^	*I*^2^ = 94%^↓^, *p* < 0.001	Yes^↑^	Yes^↓^	Intercept = 4.06^↓^, *p* = 0.02	Low
Meta-analysis 1b: odds of PLEs in adolescents with substance use *v*. adolescents without substance use								
17, 86%^↑^	102 769↑	OR = 1.77^↓^, *p* < 0.001	1.55–2.02^↑^	*I*^2^ = 57%^↓^, *p* = 0.002	Yes^↑^	Yes^↓^	Intercept = 1.10^↑^, *p* *=* 010	Moderate
Meta-analysis 2a: event rates of substance use in adolescents with PLEs								
16, 89%^↑^	3446↑	ER = 0.19, *p* = NA	0.12–0.28^↑^	*I*^2^ = 96%^↓^, *p* < 0.001	Yes^↑^	Yes^↓^	Intercept = −2.77^↑^, *p* = 0.29	Moderate to high
Meta-analysis 2b: odds of substance use in adolescents with PLEs v. adolescents without PLEs								
18, 87%^↑^	41 028↑	OR = 1.93^↓^, *p* < 0.001	1.55–2.41^↓^	*I*^2^ = 84%^↓^, *p* < 0.0001	Yes^↑^	Yes^↓^	Intercept = 1.73^↓^, *p* < 0.001	Very low

GRADE, Grading of Recommendations, Assessment, Development and Evaluation; No., number; ER, event rate; NA, not applicable to ER data; OR, odds ratio; PLEs, psychotic-like experiences; ^↑^, upgraded; ^↓^, downgraded.

All studies were observational and therefore were assumed to be of low quality (GRADE recommendation).

a Upgraded if STROBE average was ⩾75%; downgraded if STROBE average was ⩽25%.

b Upgraded if samples were large (⩾300); downgraded if samples were small (<100).

c Upgraded if effect sizes were large (ER > 0.30, OR > 5.0); downgraded if effect sizes were small (ER < 0.07, OR < 2.0).

d Upgraded if CIs were precise (ER < 0.10, OR < 0.25 in either direction from the effect size); downgraded if imprecise (ER > 0.10, OR > 0.25 in either direction from the effect size).

e Upgraded if *I*^2^ was small and *p* > 0.05; downgraded if *I*^2^ was large and *p* < 0.05.

f Upgraded if measures, samples, and comparisons (for ORs) were direct; downgraded if they were not direct.

g Upgraded if there was no residual confounding; downgraded with probable residual confounding.

h Upgraded if Eggers test *p* > 0.05; downgraded if Egger's test *p* < 0.05.

### Meta-analysis 1a: rates of PLEs among adolescents with substance use

**Fig. 2.**
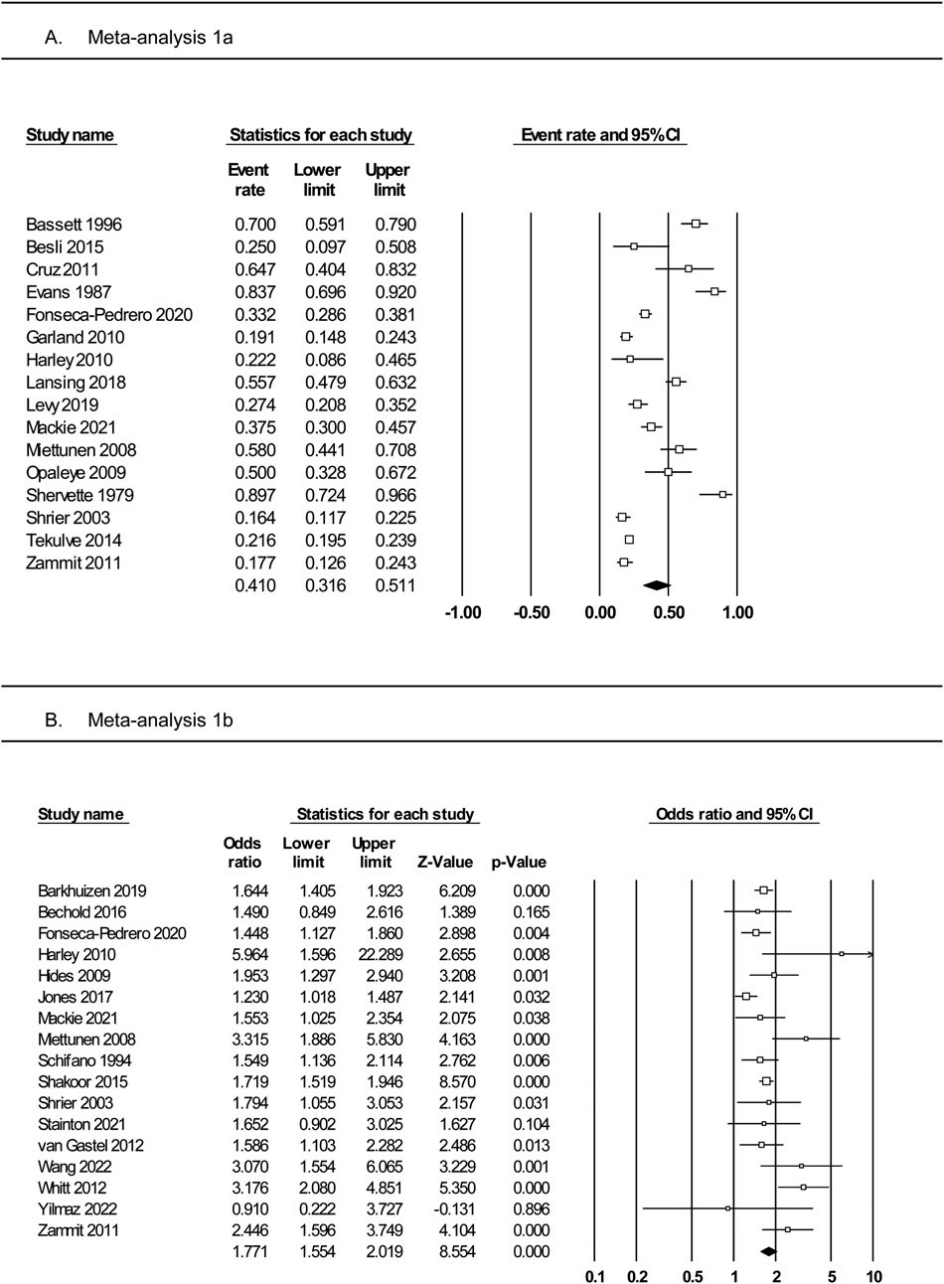
Forest plots of (panel A) prevalence rates of PLEs among adolescents with substance use and (panel B) the odds of PLEs in adolescents with *v.* without substance use.

Meta-analysis of 16 studies (Fig. 2, panel A) incorporated a total of 3050 individuals who reported using substances. The random effects model indicated that 41% of substance users reported PLEs (event rate = 0.41, 95% CI 0.32–0.51). The one-study removed analysis to assess the effects of outliers found no differences in the event rate with each study removed (rate range = 0.38–0.43). The averaged STROBE quality rating indicated a low risk of study bias (76%). The overall quality of the pooled evidence was rated ‘low’, with an imprecise CI, and substantial heterogeneity (Table 2). As Egger's test indicated possible publication bias, the adjusted event rate using Duval and Tweedie's trim and fill test was 0.27.

Subgroup analyses are presented in online Supplementary Table S3. These indicate no moderating effects of study design (cross-sectional, cohort, or chart review), substance use or PLE assessment method (interview, chart review, or self-report), type of substance used (cannabis or inhalants), whether the participant was intoxicated at the time of experiencing PLEs, or PLE type (any hallucinations, visual hallucinations, paranoia/delusions). Meta-regression analyses revealed no moderating effects of gender (% male), age at assessment, or study quality score.

### Meta-analysis 1b: odds of PLEs among adolescents with *v.* without substance use

Meta-analysis of 17 studies (Fig. 2, panel B) was conducted on a total of 102 769 individuals with and without substance use. The random effects model revealed a small-to-medium-sized effect, with adolescent substance users nearly twice as likely to report PLEs than their non-substance using counterparts (OR 1.77, 95% CI 1.55–2.02, *p* < 0.001). The one-study removed analysis found no differences in effect size with each study removed (OR range = 1.69–1.83, all *p* < 0.001). The averaged STROBE quality rating indicated a low risk of study bias (86%). The overall quality of the pooled evidence was rated ‘moderate’, with lower, but still significant heterogeneity, a precise CI, and no evidence of publication bias (Table 2).

Subgroup analyses (online Supplementary Table S4) identified no moderating effects of study design (cross-sectional, cohort, case-control, or twin study), substance use or PLE assessment method (self-report or interview), type of substance (cannabis lifetime or weekly use, alcohol, or tobacco), or type of PLE (hallucinations or paranoia/delusions). Insufficient studies reported adjusted ORs to assess this potential moderator. Meta-regression analyses also revealed no moderating effects of gender distribution or study quality. Age at assessment showed a significant medium-sized effect, with studies of younger samples having greater odds of PLEs in substance users *v.* non-users than studies with older samples (coefficient = −0.32, *p* = 0.009). Data from three studies (Lansing et al., 2018; McGorry et al., 1995; Mundy et al., 1990) were able to be pooled in a correlation meta-analysis assessing dose-dependence between the level of any substance use and the number/severity of any PLE as the outcome. This analysis (online Supplementary Fig. S5) contained 911 adolescents and found a weak but significant correlation between increased substance use and increased PLEs (correlation = 0.22, 95% CI 0.15–0.29, *p* < 0.001), with low heterogeneity (*I*^2^ = 9%).

### Meta-analysis 2a: rates of substance use among adolescents with PLEs

**Fig. 3.**
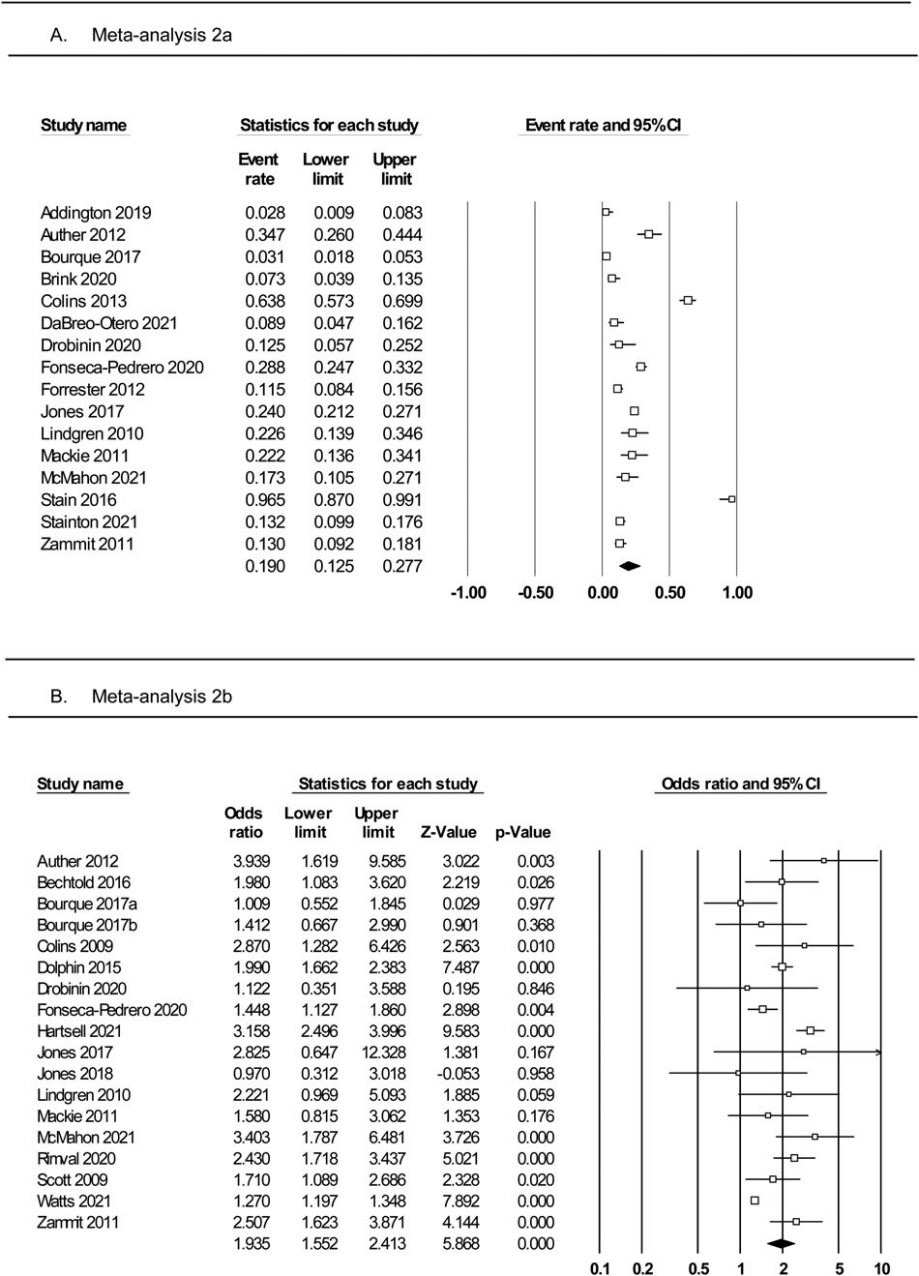
Forest plots of (panel A) prevalence rates of substance use among adolescents with PLEs and (panel B) the odds of substance use in adolescents with *v.* without PLEs.

Meta-analysis of 16 studies (Fig. 3, panel A) was conducted on a total of 3446 individuals who reported PLEs. The random effects model indicated a small effect, with 19% of individuals with PLEs reporting using substances (rate = 0.19, 95% CI 0.12–0.28). The one-study removed analysis found no differences in event rate with each study removed (rate range = 0.16–0.21). The averaged STROBE quality rating indicated a low risk of study bias (89%). The overall quality of the pooled evidence was rated ‘moderate to high’ (Table 2).

Subgroup analyses (online Supplementary Table S6) identified significant moderating effects of PLE measure (interview = 0.26, self-report = 0.11) and substance type (alcohol = 0.44, tobacco = 0.24, lifetime cannabis = 0.19, weekly cannabis = 0.04, amphetamines = 0.07, cocaine = 0.03). There were no moderating effects of study design, substance use measure, gender distribution, age at assessment, or study quality. There were insufficient studies reporting PLE type to assess this potential moderator.

### Meta-analysis 2b: odds of substance use among adolescents with and without PLEs

Meta-analysis of 18 studies (Fig. 3, panel B) was conducted on a total of 41 028 individuals with and without PLEs. The random effects model indicated a medium effect size, with adolescents who reported PLEs twice as likely to use substances compared to those not reporting PLEs (OR 1.93, 95% CI 1.55–2.41, *p* < 0.0001). The one-study removed analysis found no differences in effect size with each study removed (OR range = 1.83–2.03). The averaged STROBE quality rating indicated a low risk of study bias (87%). The quality of the pooled evidence was rated as very low (Table 2). As Egger's test indicated possible publication bias, the adjusted OR using Duval and Tweedie's trim and fill test was 1.41.

Subgroup analyses (online Supplementary Table S7) found no moderating effects of study design (cross-sectional or cohort), method of assessment of PLEs or substance use (interview or self-report), type of substance (cannabis, alcohol, tobacco, amphetamine, or cocaine), or whether ORs were adjusted. Although the analyses comparing alcohol and cocaine use between adolescents with and without PLEs were not significant, alcohol, cannabis, tobacco, and amphetamine all showed significantly increased rates of use in youth with PLEs compared to youth without PLEs. Meta-regressions revealed no moderating effects of gender, age at assessment, or study quality. There were insufficient studies reporting PLE type to assess this potential moderator. Data from three studies (DaBreo-Otero, 2021; Goulter et al., 2019; Sunderland et al., 2021) were pooled in a correlation meta-analysis assessing dose-dependence between the number/severity of any PLE and the level of any substance use as the outcome. This analysis (online Supplementary Fig. S8) contained 2995 children and adolescents and found a weak but significant correlation between increased PLEs and increased substance use (correlation = 0.17, 95% CI 0.05–0.33, *p* = 0.04), with high heterogeneity (*I*^2^ = 93%).

### Studies not included in the meta-analyses

Due to their reporting beta coefficients (Roth, Le, Oh, Van Iddekinge, & Bobko, 2018) three studies were not able to be included in the correlation meta-analyses: Albertella and Norberg (2012) reported a significant reduction in the amount of cannabis used by adolescents reporting subclinical symptoms of psychoticism and paranoid ideation on the Brief Symptom Inventory (BSI) following a 3-month residential programme [the Program for Adolescent Life Management (PALM])]. They reported no significant associations between pre-treatment psychoticism and paranoid ideation and pre-treatment cannabis use. Vaughn (2006) found that poly-substance use was related to higher mean levels of paranoid ideation and Konings et al. (2008) found that cannabis use prior to the age of 14 years, but not in the whole sample or in the sample aged over 14 years, predicted later PLEs.

Friedman et al. (1987) used adolescent non-patient BSI norms as controls so could not be combined with other controls. They reported higher mean scores on psychoticism and paranoid ideation subscales of the BSI among a sample of substance using high school students. The strength of association between substance use and psychopathology in general increased over time (from 15.1 to 16.8 years).

## Discussion

This systematic review synthesised the available evidence regarding the prevalence and association of PLEs and substance use among children and adolescents aged 17 years or younger. Results indicate that around two-in-five young people who used substances experienced PLEs (meta-analysis 1a), and around one-in-five young people who experienced PLEs reported using substances (meta-analysis 2a). Those who used substances were twice as likely to experience PLEs than those who did not use substances (meta-analysis 1b), similar to the rate of substance use in those with *v.* without PLEs (meta-analysis 2b).

Most of the included studies assessed cannabis, but alcohol, tobacco, and amphetamine use were each also associated with PLEs. These findings are consistent with prior reviews not restricted to child and adolescent samples, where 48.7% of CHR individuals reported lifetime cannabis use and 25.8% reported current cannabis use (Farris, Shakeel, & Addington, 2020), and exposure to any substance at least doubled the risk for psychotic experiences in general population samples (Linscott & van Os, 2013). We also observed a dose-dependent association between increased substance use (frequency or amount) and increased PLEs (number or severity). This has also been found among adult samples assessed for cannabis use preceding the onset of psychosis (Marconi et al., 2016), but not for PLEs when adjusted for multiple covariates (Degenhardt et al., 2018).

While heterogeneity was observed in all analyses, all but one of the subgroup analyses and meta-regressions identified no moderating effects of study design, study quality, gender, age at assessment, measure used to assess PLEs or substance use, PLE or substance type, or whether participants were intoxicated at the time of the PLE assessment. Several of these subgroup analyses were constrained by few studies, particularly those assessing PLE and substance type. A moderating effect was found in one analysis, where younger mean age at assessment was associated with increased PLEs in those with *v.* without substance use. This effect was also found in studies that assessed PLEs in early onset (defined variably as before 14 or 16 years of age) compared to late onset (14 or 16 years and older) cannabis use (Jones et al., 2017; Konings et al., 2008; Stefanis et al., 2004), indicating earlier and prolonged use increases the likelihood of PLEs. The only other moderating effect was identified in the analysis comparing rates of substance use in adolescents with and without PLEs. When PLEs were measured by interviewer rating, higher rates of substance use were observed than in studies using self-reported PLEs (rate = 0.26 *v.* 0.11). In that analysis, alcohol, tobacco, and cannabis were the most commonly used substances, and alcohol, cannabis, tobacco, and amphetamine use were each significantly greater in the subgroup analysis comparing young people with *v.* without PLEs). We also observed a dose-dependent association between increased PLEs and increased substance use, which has similarly been identified in adult samples assessing severity of PLEs and increased subsequent substance use (Degenhardt et al., 2018).

Our findings show that the rate of PLEs among substance-using youth (41%) was notably higher than the rate of PLEs identified previously in meta-analysis on samples of the general youth population (aged 13–18 years; 7.5%) (Kelleher et al., 2012). Our findings also reveal an elevated rate of substance use in adolescents experiencing PLEs (19%). Globally, between the ages of 15 and 19 years, 4.8% of males and 2.2% of females have consumed alcohol, while 2.4% of males and 1.6% of females have used illicit substances (Degenhardt et al., 2016). These relationships might be explained, in part, by a self-medication mechanism, whereby young people who experience PLEs, who may also be experiencing depression or anxiety symptoms (Varghese et al., 2011), may be at greater risk of using substances in order to cope with the potential symptom-related distress (Smit, Bolier, & Cuijpers, 2004). However, evidence to support this hypothesis is limited, and it is possible that substance misuse and PLEs share similar risk factors, such as genetic predisposition (Degenhardt & Hall, 2006). Another explanation might be that dopamine dysregulation underlies the association, as antipsychotics block dopamine receptors while agonists elicit positive symptomatology (Dean & Murray, 2005). Repeated exposure to substances that increase dopamine levels could produce a progressing and lasting response, particularly in those with a genetic predisposition (Dean & Murray, 2005). Psychotic symptoms have been shown to be elicited by progressively smaller, repeated doses of cocaine (Bartlett, Hallin, Chapman, & Angrist, 1997).

Our findings have notable clinical and policy implications. Psychotic experiences in childhood and adolescence have been associated with a four-fold increase in risk of psychotic disorders (Healy et al., 2019). The pre-prodromal phase of illness represents an opportunity for early intervention to potentially prevent and/or delay the onset of psychosis, while more benign and more effective treatments are possible (Laurens & Cullen, 2016). Substance use cessation treatment should be a focus in this early stage and included in early intervention programmes for psychotic illnesses. Considering the normative rate of substance use among youth, as well as current trends towards marijuana legalisation in many jurisdictions, increased efforts are needed to educate young people and the broader public about the serious mental health risks linked to substance use. Together, findings from the current meta-analyses suggest that delivery of universal substance use prevention programmes to youth aged 17 years and younger may help to avert PLEs, and that targeted interventions for young people with PLEs may help to discourage their engagement in substance use. These hypotheses need to be explicitly tested.

While this systematic review extends previous evidence of associations between substance use and prodromal symptoms and psychotic disorders, limitations should be noted. The analyses drew predominantly on cross-sectional data, even in the cohort studies, as many outcomes were reported >17 years of age in those studies. Therefore, the capacity to determine the direction of effects was limited. Despite assessing multiple moderators, the high levels of heterogeneity observed suggest other sources of between-study differences not investigated here. Some of the subgroup analyses were also hampered by the small number of studies, and some subgroups, such as current cannabis use and count/frequency of PLEs, were unable to be assessed due to lack of data. Access to individual participant data may allow greater assessment of between-study differences in future meta-analyses.

In summary, our findings support the notion that adolescents with PLEs have increased rates of substance use, and young substance users have increased rates of PLEs. These individuals may represent a subclinical group at risk of transitioning to CHR and psychosis, and efforts in developing early detection and intervention might prevent or postpone onset of adult psychopathology across both psychotic and addictive domains. Further rigorous longitudinal studies are needed to clarify the temporal relationship between psychosis and substance use, especially given increasing permissiveness towards recreational cannabis use.

## Supplementary material

For supplementary material accompanying this paper visit https://doi.org/10.1017/S0033291722003440. click here to view supplementary material
