# Supplementary material for: Substance use and psychotic-like experiences in young people: a systematic review and meta-analysis
Source: Psychol Med. 2022 Nov 15;53(2):305–19. doi: 10.1017/S0033291722003440 (PMC9899577; doi:10.1017/S0033291722003440)
Supplement: Supplementary file 1 [file S0033291722003440sup001.docx]

**Supplementary Material**

S1. Text. Systematic review search terms

S2. Table. Results of the Strengthening the Reporting of Observational Studies in Epidemiology [STROBE] Checklist

S3. Table. Moderator analyses for meta-analysis 1a: Rates of psychotic-like experiences in adolescents with substance use

S4. Table. Moderator analyses for meta-analysis 1b: Odds of psychotic-like experiences in adolescents with versus without substance use

S5. Figure. Correlation meta-analysis assessing dose-dependence between the level of substance use (alcohol, cannabis, or any substance) and the number/severity of any PLE

S6. Table. Moderator analysis for meta-analysis 2a: Rates of substance use in adolescents with psychotic-like experiences

S7. Table. Moderator analyses for meta-analysis 2b: Odds of substance use in adolescents with versus without psychotic-like experiences

S8. Figure. Correlation meta-analysis assessing dose-dependence between the level of any PLE and the number/severity of any substance use

S1. Supplementary text. Systematic review search terms

*(Population terms)* adolescen* OR teen* OR young* OR youth* OR juvenile* OR child* OR offspring* OR p#diatric* or kid* OR minor OR boy* OR girl* OR preschool* OR pre-school* OR “school age*” OR schoolchild*

AND

*(Psychotic-like experience terms)* hallucination* OR delusion* OR “attenuated psycho*” OR “psychotic symptom*” OR “psychosis symptom*” OR “psychotic experience*” OR “psychosis experience*” OR “psychotic-like” OR “psychosis-like” OR paranoi* OR prodrom*

AND

*(Substance use terms)* "Illicit drug*" OR "Illegal Drug*" OR "Substance* use" OR "Substance* misuse" OR "Substance* abuse" OR Cannab* OR Hemp OR Weed OR THC OR Tetrahydrocannabinol OR Marijuana OR Tobacco OR Cigarette OR Smok* OR Alcohol OR "Binge drink*" OR Amphetamine* OR Meth OR Methamphetamin* OR "Crystal Meth" OR Ice OR Ecstasy OR MDMA OR Molly OR Cocaine OR Crack OR Opiat* OR Opioid* OR *LSD* OR "Lysergic acid diethylamide" OR Hallucinogen* OR Inhalant*

S2. Table. Results of the Strengthening the Reporting of Observational Studies in Epidemiology [STROBE] Checklist

|  | Item Number | | | | | | | | | | | | | | | | | | | | | | | | | | | | | | | | | |  |
| --- | --- | --- | --- | --- | --- | --- | --- | --- | --- | --- | --- | --- | --- | --- | --- | --- | --- | --- | --- | --- | --- | --- | --- | --- | --- | --- | --- | --- | --- | --- | --- | --- | --- | --- | --- |
| Study Name | 1a | 1b | 2 | 3 | 4 | 5 | 6a | 6b | 7 | 8 | 9 | 10 | 11 | 12a | 12b | 12c | 12d | 12e | 13a | 13b | 13c | 14a | 14b | 14c | 15 | 16a | 16b | 16c | 17 | 18 | 19 | 20 | 21 | 22 | Total% |
| Addington 2019 | 1 | 1 | 1 | 1 | 1 | 1 | 1 | - | 1 | 1 | 1 | 1 | 1 | 1 | 1 | - | - | - | 1 | - | - | 1 | - | - | 1 | 1 | - | - | 1 | 1 | 1 | 1 | 0 | 1 | 96% |
| Albertella 2012 | 0 | 1 | 1 | 1 | 1 | 1 | 1 | - | 1 | 1 | 0 | 1 | 1 | 1 | 1 | 0 | - | - | 0 | 0 | 0 | 1 | 1 | 1 | 1 | 1 | - | - | 1 | 1 | 1 | 1 | 1 | 0 | 76% |
| Auther 2012 | 1 | 1 | 1 | 1 | 1 | 1 | 1 | 1 | 1 | 1 | 1 | 0 | 1 | 1 | 1 | 0 | 1 | - | 0 | 1 | 0 | 1 | 0 | - | 1 | 1 | 1 | - | 1 | 1 | 1 | 1 | 1 | 1 | 84% |
| Barkhuizen 2019 | 1 | 1 | 1 | 1 | 1 | 1 | 1 | 1 | 1 | 1 | 1 | 1 | 1 | 1 | 1 | 0 | 1 | 1 | 1 | 1 | 0 | 1 | 0 | - | 1 | 1 | 1 | - | 1 | 1 | 1 | 1 | 1 | 1 | 91% |
| Bassett 1996 | 1 | 1 | 0 | 1 | 1 | 1 | 1 | - | 0 | 1 | 1 | 1 | 1 | 0 | 0 | 0 | - | - | 1 | 1 | 0 | 1 | 1 | - | 1 | 0 | - | - | 1 | 1 | 0 | 1 | 1 | 0 | 68% |
| Bechtold 2016 | 0 | 1 | 1 | 1 | 1 | 1 | 1 | - | 1 | 1 | 1 | 1 | 1 | 1 | 1 | 1 | - | 1 | 1 | 1 | 0 | 1 | 1 | 1 | 1 | 1 | 1 | - | 1 | 1 | 1 | 1 | 1 | 1 | 94% |
| Besli 2015 | 1 | 1 | 1 | 1 | 1 | 1 | 1 | - | 0 | 1 | 0 | 1 | 0 | 0 | 0 | 0 | - | - | 0 | 1 | 0 | 1 | 1 | - | 1 | 1 | - | - | - | 1 | 1 | 1 | 1 | 0 | 67% |
| Bourque 2017a | 0 | 1 | 1 | 1 | 1 | 1 | 1 | - | 1 | 1 | 1 | 1 | 1 | 1 | 1 | 1 | - | - | 1 | 1 | 0 | 1 | 0 | 1 | 1 | 1 | 1 | - | 1 | 1 | 1 | 1 | 1 | 1 | 90% |
| Bourque 2017b | 0 | 1 | 1 | 1 | 1 | 1 | 1 | 1 | 1 | 1 | 1 | 1 | 1 | 1 | - | 0 | 1 | 1 | 1 | 1 | 0 | 1 | 0 | - | 1 | 1 | - | - | 1 | 1 | 1 | 1 | 1 | 1 | 87% |
| Brink 2020 | 1 | 1 | 1 | 1 | 1 | 1 | 1 | - | 1 | 1 | 1 | 1 | 1 | 1 | - | 1 | 1 | 1 | 1 | 1 | 0 | 1 | 1 | 1 | 1 | 1 | 1 | - | 1 | 1 | 1 | 1 | 1 | 1 | 97% |
| Colins 2009 | 0 | 1 | 1 | 1 | 1 | 1 | 1 | - | 1 | 1 | 1 | 1 | 1 | 1 | 1 | 0 | - | 1 | 1 | 1 | 0 | 1 | 0 | - | 1 | 1 | 1 | - | 1 | 1 | 1 | 1 | 1 | 1 | 87% |
| Colins 2013 | 0 | 1 | 1 | 1 | 1 | 1 | 1 | - | 1 | 1 | 1 | 1 | 1 | 1 | 1 | 0 | - | - | 1 | 1 | 0 | 1 | - | - | 1 | 1 | - | - | 1 | 1 | 1 | 1 | 1 | 0 | 85% |
| Cruz 2011 | 1 | 1 | 1 | 1 | 1 | 1 | 1 | - | 1 | 1 | 0 | 1 | - | - | - | - | - | - | 1 | 1 | 0 | 1 | - | - | 1 | - | - | - | - | 1 | 1 | 0 | 1 | 0 | 81% |
| DaBreo Otero 2021 | 0 | 1 | 1 | 1 | 1 | 1 | 1 | - | 1 | 1 | 1 | 1 | 1 | 1 | - | 1 | 0 | - | 0 | 0 | 0 | 1 | 1 | 1 | 1 | 1 | - | - | - | 1 | 1 | 1 | 1 | - | 81% |
| Dolphin 2015 | 1 | 1 | 1 | 1 | 1 | 1 | 1 | - | 1 | 1 | 1 | 1 | 1 | 1 | 1 | 1 | 1 | - | 1 | 0 | 0 | 1 | 1 | - | 1 | 1 | 1 | - | 1 | 1 | 1 | 1 | 1 | 0 | 90% |
| Drobinin 2020 | 1 | 1 | 1 | 1 | 1 | 1 | 0 | - | 1 | 1 | 1 | 0 | 1 | 1 | - | - | - | 1 | 1 | - | - | 1 | - | - | 1 | 1 | 1 | - | 1 | 1 | 1 | 1 | 1 | 1 | 92% |
| Evans 1987 | 0 | 1 | 1 | 1 | 1 | 1 | 1 | - | 1 | 1 | 0 | 1 | 0 | 0 | 0 | 0 | - | - | 0 | - | - | 0 | - | - | 1 | 1 | - | - | 1 | 0 | 0 | 1 | 0 | 0 | 52% |
| Fonseca-Pedrero 2020 | 0 | 1 | 1 | 1 | 1 | 1 | 1 | - | 1 | 1 | 1 | 1 | 1 | 1 | 1 | 0 | - | - | 1 | 1 | 0 | 1 | 0 | - | 1 | 1 | 1 | - | 1 | 1 | 1 | 1 | 1 | 1 | 86% |
| Forrester 2012 | 0 | 1 | 1 | 1 | 1 | 1 | 1 | - | 1 | 1 | 0 | 1 | 1 | 1 | - | 0 | - | - | 1 | - | - | 1 | - | - | 1 | 1 | - | - | - | 1 | 1 | 1 | 1 | 1 | 87% |
| Friedman 1987 | 1 | 1 | 1 | 0 | 1 | 1 | 1 | - | 1 | 1 | 0 | 1 | 1 | 1 | 1 | 0 | - | - | 0 | - | - | 1 | 1 | 1 | 1 | 0 | - | - | 1 | 0 | 1 | 1 | 0 | 0 | 70% |
| Garland 2010 | 0 | 1 | 1 | 1 | 1 | 1 | 1 | - | 1 | 1 | 1 | 1 | 1 | 1 | 1 | 0 | 0 | - | 1 | 1 | 0 | 1 | 0 | - | 1 | 1 | 1 | - | 1 | 1 | 1 | 1 | 1 | 0 | 80% |
| Goulter 2019 | 0 | 1 | 1 | 1 | 1 | 1 | 1 | - | 1 | 1 | 1 | 1 | 1 | 1 | - | 1 | - | - | 0 | 0 | 0 | 0 | 0 | 0 | 1 | 1 | - | - | 1 | 1 | 1 | 1 | 1 | 1 | 75% |
| Harley 2010 | 0 | 1 | 1 | 1 | 1 | 1 | 1 | 1 | 1 | 1 | 1 | 1 | 1 | 1 | 1 | 0 | 1 | - | 1 | 1 | 0 | 0 | 0 | - | 1 | 1 | 1 | - | 1 | 1 | 1 | 1 | 1 | 1 | 84% |
| Hartsell 2021 | 1 | 1 | 1 | 1 | 1 | 1 | 1 | - | 1 | 1 | 1 | 1 | 1 | 1 | - | 1 | 0 | - | 0 | 0 | 0 | 1 | 1 | 1 | 1 | 1 | 1 | - | 1 | 1 | 1 | 1 | 1 | 0 | 83% |
| Hides 2009 | 0 | 1 | 1 | 1 | 1 | 1 | 1 | - | 1 | 1 | 1 | 1 | 1 | 1 | 1 | 0 | 0 | - | 1 | 1 | 0 | 1 | 0 | - | 1 | 1 | 1 | - | 1 | 1 | 1 | 1 | 1 | 1 | 83% |
| Jones 2017 | 1 | 1 | 1 | 1 | 1 | 1 | 1 | - | 1 | 1 | 1 | 1 | 1 | 1 | 1 | 0 | 0 | - | 1 | 1 | 0 | 1 | 1 | - | 1 | 1 | 1 | - | 1 | 1 | 1 | 1 | 1 | 1 | 90% |
| Jones 2018 | 1 | 1 | 1 | 1 | 1 | 1 | 1 | - | 1 | 1 | 1 | 1 | 1 | 1 | 1 | 1 | 0 | 1 | 1 | 1 | 0 | 1 | 1 | 1 | 0 | 1 | 1 | - | 1 | 1 | 1 | 1 | 1 | 1 | 91% |
| Konings 2008 | 0 | 1 | 1 | 1 | 1 | 1 | 1 | - | 1 | 1 | 1 | 1 | 1 | 1 | - | 1 | - | 1 | 1 | - | - | 1 | 0 | - | 1 | 1 | - | - | 1 | 1 | 1 | 0 | 1 | 0 | 85% |
| Lansing 2018 | 0 | 1 | 1 | 1 | 1 | 1 | 1 | - | 1 | 1 | 1 | 1 | 1 | 1 | 1 | 1 | 1 | - | 1 | 1 | 0 | 1 | 1 | - | 1 | 1 | 1 | - | 1 | 1 | 1 | 1 | 1 | 1 | 93% |
| Levy 2019 | 0 | 0 | 0 | 1 | 1 | 1 | 1 | - | 1 | 1 | 1 | 1 | 1 | 1 | 1 | 0 | - | - | 1 | - | - | 1 | 0 | - | 1 | 1 | 1 | - | 1 | 1 | 1 | 1 | 1 | 1 | 81% |
| Lindgren 2010 | 0 | 1 | 1 | 1 | 1 | 1 | 1 | 1 | 0 | 0 | 1 | 1 | 1 | 1 | 1 | 1 | 0 | - | 1 | 0 | 0 | 1 | 1 | - | 1 | 1 | 1 | - | 1 | 1 | 1 | 1 | 1 | 1 | 81% |
| Mackie 2011 | 0 | 1 | 1 | 1 | 1 | 1 | 1 | - | 1 | 1 | 1 | 1 | 1 | 1 | 1 | - | 1 | - | 1 | 1 | 0 | 1 | - | 1 | 1 | 1 | 1 | - | 1 | 1 | 1 | 1 | 1 | 1 | 93% |
| Mackie 2021 | 0 | 1 | 1 | 1 | 1 | 1 | 1 | - | 1 | 1 | 1 | 1 | 1 | 1 | 1 | - | - | 1 | 1 | 1 | 0 | 1 | - | - | 1 | 1 | 1 | - | 1 | 1 | 1 | 1 | 1 | 1 | 93% |
| McGorry 1995 | 1 | 1 | 1 | 1 | 1 | 1 | 1 | - | 1 | 1 | 1 | 1 | 1 | 1 | 1 | 0 | - | - | 0 | 1 | 0 | 1 | 1 | - | 1 | 0 | - | - | 1 | 1 | 1 | 1 | 1 | 1 | 86% |
| McMahon 2021 | 1 | 1 | 1 | 1 | 1 | 1 | 1 | - | 1 | 1 | 1 | 1 | 1 | 1 | - | 1 | - | - | 1 | 0 | 0 | 1 | 1 | 1 | 1 | 1 | 1 | - | 1 | 1 | 1 | 1 | 1 | 1 | 93% |
| Miettunen 2008 | 1 | 1 | 1 | 1 | 1 | 1 | 1 | - | 1 | 1 | 1 | 1 | 1 | 0 | 0 | 0 | - | 1 | 1 | 1 | 0 | 1 | 0 | - | 1 | 1 | 1 | - | 1 | 1 | 0 | 1 | 1 | 1 | 80% |
| Mundy 1990 | 0 | 1 | 1 | 1 | 1 | 1 | 1 | - | 1 | 1 | 1 | 1 | 1 | 1 | - | - | - | - | 1 | 1 | 0 | 1 | - | - | 1 | 1 | - | - | 1 | 1 | 1 | 0 | 1 | 1 | 88% |
| Opaleye 2009 | 0 | 1 | 1 | 1 | 1 | 1 | 1 | - | 1 | 1 | 1 | 1 | 1 | 1 | - | - | - | - | 1 | 1 | 0 | 1 | - | - | 1 | 1 | - | - | - | 1 | 1 | 1 | 1 | 1 | 92% |
| Rimval 2020 | 1 | 1 | 1 | 1 | 1 | 1 | 1 | - | 1 | 1 | 1 | 1 | 1 | 1 | - | 0 | - | 1 | 1 | 0 | 0 | 1 | 1 | 1 | 0 | 0 | 1 | - | 1 | 1 | 1 | 1 | 1 | 1 | 83% |
| Schifano 1994 | 0 | 1 | 1 | 1 | 1 | 1 | 1 | - | 1 | 1 | 1 | 1 | 1 | 1 | 0 | 0 | - | - | 1 | 1 | 0 | 1 | 0 | - | 1 | 1 | 1 | - | 1 | 1 | 1 | 1 | 1 | 1 | 83% |
| Scott 2009 | 1 | 1 | 1 | 1 | 1 | 1 | 1 | - | 1 | 1 | 1 | 0 | 1 | 1 | 1 | 0 | 1 | - | 0 | 0 | 0 | 1 | 0 | - | 1 | 1 | 1 | - | 1 | 1 | 1 | 1 | 1 | 1 | 80% |
| Shakoor 2015 | 1 | 1 | 1 | 1 | 1 | 1 | 1 | - | 1 | 1 | 1 | 1 | 1 | 1 | 1 | 0 | - | - | 1 | 1 | 0 | 1 | 0 | - | 1 | 1 | 1 | - | 1 | 1 | 1 | 1 | 1 | 1 | 90% |
| Shervette 1979 | 0 | 1 | 1 | 0 | 1 | 1 | 1 | - | 0 | 1 | 0 | 1 | 0 | 0 | - | - | - | - | 0 | 0 | 0 | 1 | 1 | - | 1 | - | - | - | - | 0 | 0 | 1 | 0 | 0 | 46% |
| Shrier 2003 | 0 | 1 | 1 | 1 | 1 | 1 | 1 | - | 1 | 1 | 1 | 0 | 1 | 1 | 1 | 0 | - | 0 | 0 | 0 | 0 | 1 | 0 | - | 1 | 1 | 0 | - | 1 | 1 | 1 | 1 | 1 | 1 | 70% |
| Stain 2016 | 1 | 1 | 1 | 1 | 1 | 1 | 1 | - | 1 | 1 | 1 | 1 | 1 | 1 | - | 1 | - | 1 | 1 | 1 | 1 | 1 | 1 | 1 | 1 | 1 | - | - | 1 | 1 | 1 | 1 | 1 | 1 | 100% |
| Stainton 2021 | 0 | 1 | 1 | 1 | 1 | 1 | 1 | - | 1 | 1 | 1 | 0 | 1 | 1 | 1 | 1 | - | - | 0 | 0 | 0 | 1 | 0 | - | 1 | 1 | - | - | 1 | 1 | 1 | 1 | 1 | 1 | 79% |
| Sunderland 2021 | 1 | 1 | 1 | 1 | 1 | 1 | 1 | - | 1 | 1 | 1 | 1 | 1 | 1 | 1 | - | 1 | - | 0 | 0 | 0 | 1 | - | - | 1 | 1 | - | - | 1 | 1 | 1 | 1 | 1 | 0 | 85% |
| Tekulve 2014 | 0 | 1 | 1 | 1 | 1 | 1 | 1 | - | 1 | 1 | 0 | 1 | 0 | 0 | 0 | 0 | - | 0 | 0 | 0 | 0 | 1 | 0 | 0 | 1 | 0 | - | - | 1 | 1 | 1 | 1 | 1 | 0 | 53% |
| van Gastel 2012 | 1 | 1 | 1 | 1 | 1 | 1 | 1 | - | 1 | 1 | 1 | 1 | 1 | 1 | 1 | 0 | - | 1 | 0 | 0 | 0 | 1 | 0 | 0 | 1 | 1 | 1 | - | 1 | 1 | 1 | 1 | 1 | 0 | 77% |
| Vaughn 2006 | 0 | 1 | 1 | 1 | 1 | 1 | 1 | - | 1 | 1 | 1 | 1 | 1 | 1 | 1 | 1 | - | 0 | 1 | 1 | 0 | 1 | - | - | 1 | 1 | 1 | - | 1 | 1 | 1 | 1 | 1 | 0 | 86% |
| Wang 2022 | 1 | 1 | 1 | 1 | 1 | 1 | 1 | - | 1 | 1 | 1 | 1 | 1 | 1 | - | - | - | - | 1 | 1 | 0 | 1 | - | - | 1 | 1 | - | - | - | 1 | 1 | 1 | 1 | 1 | 96% |
| Watts 2021 | 0 | 1 | 1 | 1 | 1 | 1 | 1 | - | 1 | 1 | 1 | 0 | 1 | 1 | - | 0 | - | - | 0 | 0 | 0 | 1 | 0 | - | 1 | 1 | - | - | - | 1 | 1 | 1 | 1 | 1 | 73% |
| Whitt 2012 | 0 | 1 | 1 | 1 | 1 | 1 | 1 | - | 1 | 1 | 1 | 0 | 1 | 1 | 1 | 0 | - | - | 0 | 0 | 0 | 1 | 0 | - | 1 | 1 | 1 | - | 1 | 1 | 1 | 1 | 1 | 1 | 76% |
| Yilmaz 2022 | 1 | 1 | 1 | 1 | 1 | 1 | 1 | - | 1 | 1 | 1 | 1 | 1 | 1 | - | 1 | - | - | 1 | 1 | 0 | 1 | 1 | - | 1 | 1 | - | - | - | 1 | 1 | 1 | 1 | 1 | 96% |
| Zammit 2011 | 1 | 1 | 1 | 1 | 1 | 1 | 1 | - | 1 | 1 | 1 | 0 | 1 | 1 | 1 | 1 | - | 1 | 1 | 1 | 0 | 1 | 1 | 1 | 1 | 1 | 1 | - | 1 | 1 | 1 | 1 | 1 | 1 | 94% |

Total % = percentage of checklist items within each observational study; Dash (-) = a non-applicable item which has been omitted from the total percentage count

S3. Table Moderator analyses for meta-analysis 1a: Rates of psychotic-like experiences in adolescents with substance use

| Subgroup analyses | Studies | Total N | Event rate (95%CIs) |
| --- | --- | --- | --- |
| Study design |  |  |  |
| Cross-sectional | 9 | 1365 | 0.40 (0.29 to 0.53) |
| Chart review | 4 | 1453 | 0.53 (0.19 to 0.84) |
| Cohort | 2 | 214 | 0.35 (0.08 to 0.77) |
| Substance measure |  |  |  |
| Interview | 10 | 1236 | 0.38 (0.27 to 0.51) |
| Chart review | 3 | 1437 | 0.62 (0.20 to 0.92) |
| Self-report | 3 | 377 | 0.35 (0.16 to 0.60) |
| PLE measure |  |  |  |
| Interview | 7 | 716 | 0.43 (0.24 to 0.64) |
| Self-report | 5 | 881 | 0.33 (0.24 to 0.43) |
| Chart review | 4 | 1453 | 0.53 (0.19 to 0.84) |
| Intoxication effects |  |  |  |
| Intoxicated | 9 | 1956 | 0.49 (0.33 to 0.65) |
| Non-intoxicated | 7 | 1094 | 0.33 (0.22 to 0.46) |
| Substance type |  |  |  |
| Cannabis - lifetime use | 6 | 899 | 0.32 (0.24 to 0.42) |
| Inhalants - current use | 2 | 310 | 0.52 (0.05 to 0.96) |
| PLE type |  |  |  |
| Hallucinations (any) | 11 | 948 | 0.49 (0.35 to 0.64) |
| Visual hallucinations | 2 | 310 | 0.62 (0.03 to 0.99) |
| Paranoia/delusions | 2 | 201 | 0.77 (0.22 to 0.98) |
| Meta-regressions | Studies | Total N | Coefficient (95%CIs) |
| % male | 13 | 2825 | 0.00 (-0.03 to 0.03) |
| Mean age at assessment | 13 | 2838 | -0.84 (-1.99 to 0.32) |
| Study quality score | 16 | 3050 | -0.03 (-0.07 to 0.01) |

All p-values for the Q_B_ (between subgroups) statistic analyses were >0.05

PLE type subgroups contain non-independent samples

S4. Table. Moderator analyses for meta-analysis 1b: Odds of psychotic-like experiences in adolescents with versus without substance use

| Subgroup analyses | Studies | Total N | OR (95%CIs) |
| --- | --- | --- | --- |
| Study design |  |  |  |
| Cross-sectional | 10 | 78 134 | 1.77 (1.50 to 2.09)*** |
| Cohort | 3 | 13 136 | 2.07 (1.09 to 3.92)* |
| Case-control | 2 | 2 825 | 2.37 (0.38 to 14.96) |
| Twin study | 2 | 13 447 | 1.69 (1.53 to 1.86)*** |
| Substance measure |  |  |  |
| Self-report | 11 | 94 067 | 1.69 (1.43 to 1.99)*** |
| Interview | 6 | 12 525 | 1.96 (1.52 to 2.53)*** |
| PLE measure |  |  |  |
| Self-report | 11 | 70 993 | 1.77 (1.54 to 2.03)*** |
| Interview | 6 | 14 003 | 1.81 (1.37 to 2.39)*** |
| Substance type |  |  |  |
| Cannabis - lifetime use | 11 | 31 541 | 1.71 (1.45 to 2.01)*** |
| Cannabis - weekly use | 6 | 19 229 | 2.27 (1.31 to 3.95)** |
| Tobacco - current use | 4 | 74 513 | 1.72 (1.43 to 2.06)*** |
| Alcohol - lifetime use | 2 | 70 162 | 2.30 (1.22 to 4.34)* |
| PLE type |  |  |  |
| Hallucinations | 7 | 16 692 | 1.74 (1.54 to 1.97)*** |
| Paranoia/delusions | 7 | 17 069 | 1.92 (1.70 to 2.17)*** |
| Meta-regressions | Studies | Total N | Coefficient (95%CIs) |
| % male | 15 | 100 165 | 0.00 (-0.00 to 0.01) |
| Mean age at assessment | 11 | 93 281 | -0.32 (-0.53 to -0.10)** |
| Study quality score | 17 | 102 769 | -0.01 (-0.03 to 0.01) |

All p-values for the Q_B_ (between subgroups) statistic analyses were >0.05

Substance and PLE type subgroups contain non-independent samples

There were insufficient studies reporting adjusted ORs to conduct subgroup analysis

**p*<0.05, ***p*<0.01, ****p*<0.001

S5. Figure. Correlation meta-analysis assessing dose-dependence between the level of any substance use and the number/severity of any PLE

S6. Table. Moderator analysis for meta-analysis 2a: Rates of substance use in adolescents with psychotic-like experiences

| Subgroup analyses | Studies | Total N | Event rate (95%CIs) |
| --- | --- | --- | --- |
| Study design |  |  |  |
| Cohort | 9 | 2157 | 0.15 (0.07 to 0.28) |
| Cross-sectional | 4 | 865 | 0.18 (0.10 to 0.29) |
| Substance measure |  |  |  |
| Interview | 7 | 701 | 0.29 (0.12 to 0.56) |
| Self-report | 8 | 2440 | 0.14 (0.09 to 0.21) |
| PLE measure* |  |  |  |
| Interview | 10 | 2158 | 0.26 (0.16 to 0.39) |
| Self-report | 5 | 983 | 0.11 (0.05 to 0.19) |
| Substance type^***^ |  |  |  |
| Cannabis - lifetime use | 14 | 3079 | 0.19 (0.12 to 0.29) |
| Cannabis - weekly use | 2 | 1023 | 0.04 (0.00 to 0.28) |
| Alcohol | 9 | 1658 | 0.44 (0.33 to 0.56) |
| Tobacco | 7 | 1617 | 0.24 (0.11 to 0.44) |
| Amphetamines | 2 | 149 | 0.07 (0.02 to 0.21) |
| Cocaine | 3 | 265 | 0.03 (0.01 to 0.09) |
| Meta-regressions | Studies | Total N | Coefficient (95%CIs) |
| % male | 15 | 3223 | 0.01 (-0.04 to 0.06) |
| Mean age at assessment | 15 | 3223 | 0.39 (-0.50 to 1.27) |
| Study quality score | 16 | 3446 | 0.02 (-0.12 to 0.17) |

Substance type subgroups contain non-independent samples

There were insufficient studies reporting PLE type to conduct subgroup analysis

**p*<0.05, ****p*<0.001 for Q_B_

S7. Table. Moderator analyses for meta-analysis 2b: Odds of substance use in adolescents with versus without psychotic-like experiences

| Subgroup analyses | Studies | Total N | OR (95%CIs) |
| --- | --- | --- | --- |
| Study design |  |  |  |
| Cross-sectional | 8 | 22 769 | 1.76 (1.37 to 2.25)*** |
| Cohort | 9 | 17 927 | 2.09 (1.55 to 2.82)*** |
| Substance measure |  |  |  |
| Interview | 7 | 13 023 | 2.12 (1.77 to 2.53)*** |
| Self-report | 11 | 28 005 | 1.84 (1.38 to 2.45)*** |
| PLE measure |  |  |  |
| Interview | 11 | 21 611 | 1.98 (1.64 to 2.39)*** |
| Self-report | 7 | 19 259 | 1.85 (1.22 to 2.80)*** |
| Substance type |  |  |  |
| Cannabis - lifetime use | 14 | 27 746 | 1.99 (1.59 to 2.49)*** |
| Cannabis - weekly use | 4 | 8987 | 2.25 (1.17 to 4.32)* |
| Alcohol | 7 | 23 245 | 1.43 (1.13 to 1.82)** |
| Tobacco | 6 | 9728 | 1.95 (1.06 to 3.58)* |
| Amphetamines | 3 | 501 | 2.28 (1.23 to 4.24)** |
| Cocaine | 3 | 958 | 1.78 (0.95 to 3.36) |
| ORs |  |  |  |
| Adjusted | 4 | 15 188 | 2.09 (1.18 to 3.68)* |
| Non-adjusted | 4 | 12 702 | 1.95 (1.66 to 2.29)*** |
| Meta-regressions | Studies | Total N | Coefficient (95%CIs) |
| % male | 17 | 38 398 | 0.01 (-0.00 to 0.02) |
| Mean age at assessment | 16 | 37 515 | 0.19 (-0.01 to 0.19) |
| Study quality score | 18 | 41 028 | 0.01 (-0.02 to 0.04) |

All p-values for the Q_B_ (between subgroups) statistic analyses were >0.05

Substance and PLE type subgroups contain non-independent samples

There were insufficient studies reporting PLE type to conduct subgroup analysis

**p*<0.05, ***p*<0.01, ****p*<0.001

S8. Figure. Correlation meta-analysis assessing dose-dependence between the number/severity of any PLE and the level of any substance use
